# Gastrointestinal trichobezoars in the pediatric population: a retrospective study

**DOI:** 10.1186/s12887-023-04489-x

**Published:** 2024-02-16

**Authors:** Linyan Wang, Yi Chen, Sai Chen, Zhigang Gao, Yunzhong Qian, Qingjiang Chen

**Affiliations:** Department of General Surgery, Children’s Hospital, Zhejiang University School of Medicine, National Clinical Research Center For Child Health, Hangzhou, 310052 China

**Keywords:** Trichobezoar, Gastric trichobezoar, Intestinal obstruction, Laparoscopic, Minilaparotomy

## Abstract

**Background:**

Trichobezoar is an extremely rare condition characterized by a foreign body in the gastrointestinal tract (GIT) among children. The foreign body may exist in the digestive tract for several years, and it becomes evident if complications develop. The current study aimed to present 21 cases of GIT trichobezoars.

**Methods:**

Retrospective analysis of children who were diagnosed with trichobezoars between August 2012 and December 2022. Patient demographics, clinical presentation, diagnosis, and therapy were collected and analyzed.Twenty-one patients had GIT trichobezoars. Data were collected and analyzed retrospectively.

**Results:**

Twenty-one patients were identified. All patients were female. Their mean age at admission was 8.9 ± 1.9 years. Furthermore, 19 (90.5%) patients presented with abdominal pain, 16 (76.2%) with vomiting, and 13 (61.9%) with a palpable mass. Sixteen patients underwent gastroduodenoscopy. Among them, 15 had gastric trichobezoars. Moreover, 12 patients underwent computed tomography scan. Eight patients presented with gastric and small intestinal BZs, one presented with increased small intestinal contents with dilation, and one presented with abundant gastric contents. Then, 20 patients underwent surgery. Among them, five underwent laparoscopic-assisted minilaparotomy (LAML), and the rest underwent laparotomy. The results showed that 10 (50%) patients had gastric trichobezoars; 7 (35%), Rapunzel syndrome; and 3 (15%), small bowel trichobezoars. Two patients developed superficial wound infection postoperatively. One patient had a recurrent gastric trichobezoar.

**Conclusion:**

Trichobezoar should be considered in young girls with a history of hair eating or those with hair in the vomit or feces. Timely diagnosis and aggressive treatment are the keys to reducing complications and improving prognosis. Laparoscopic-assisted minilaparotomy is a safe, feasible, and effective surgical method for treating trichobezoars.

## Introduction

Bezoars (BZs) are conglomerations of undigested foreign materials in the gastrointestinal tract (GIT). According to their composition, BZs are classified into phytobezoar (vegetable matter), trichobezoar (hair), lactobezoar (milk protein/curd), and pharmacobezoars (medications) [1]. GIT trichobezoars are extremely rare, accounting for approximately 6% of all BZ cases.

Trichobezoars are most commonly observed in children and adolescents and are often associated with underlying psychiatric disorders such as trichotillomania and trichophagia. They are typically found in the stomach, and some may occasionally extend into the jejunum, ileum, and colon as a tail, which is referred to as Rapunzel syndrome.

In the early stage, several patients may be asymptomatic. Trichobezoar may exist in the digestive tract, which has not been identified for several years, and it becomes evident with the development of complications. Patients with trichobezoar may present with obstruction, gastrointestinal perforation, intussusception, digestive bleeding, pancreatitis, appendicitis, obstructive jaundice, and protein-losing enteropathy, and may even die [2, 3].

There are several related reports on trichobezoar. However, most are case reports or small case series. Therefore, the current study retrospectively analyzed the clinical data of 21 patients with GIT trichobezoars admitted to our hospital within the last 10 years. Moreover, it aims to discuss the case presentation, diagnosis, and therapy.

## Methods

A retrospective study was performed by retrieving the medical records of patients with trichobezoars managed at our center between August 2012 and December 2022. Inclusion criteria: Children diagnosed with trichobezoars via gastroduodenoscopy and/or surgery, or considered for bezoars via other imaging. Exclusion criteria: No trichobezoar was found during the surgery. Data including demographic characteristics, clinical presentation, diagnostic methods, treatment, postoperative complications, and final outcome were collected and analyzed. The authors assert that all the procedures in this study conformed and approved by Ethics Committee of the Children’s Hospital, Zhejiang University School of Medicine.

### Statistical analysis

Data were shown as the medians ± IQRs or mean ± standard error (SEM) depending on data characteristics. Statistical analysis was performed with SPSS18.0 and Graphpad Prism 6. Statistical *p*-values were analyzed by a two-tailed Student’s t-test. *P*-values < 0.05 were considered statistically significant.

## Results

All patients (N = 21) were female. Their mean age at admission was 8.9 ± 1.9 (range: 3.5–11.9) years, and their mean weight was 27.1 ± 8.6 (range: 10–40) kg.

The common clinical manifestations included abdominal pain, nausea and vomiting, and palpable mass (Table 1). Twelve (57.1%) patients had trichophagia. Among them, five had hair in the feces. The duration of trichophagia ranged from 1 to 6 years. However, two patients stopped ingesting hair after 3 and 6 years. One patient vomited several times with hair, and two patients had hair in the feces. Three (14.3%) patients experienced acute intestinal obstruction due to small intestinal trichobezoars.

**Table 1:** Clinical characteristics of the patients

Clinical manifestations	N (%)
Abdominal pain	19 (90.5%)
Nausea and vomiting	16 (76.2%)
Palpable mass	13 (61.9%)
Abdominal distension	4 (19.0%)
Constipation	6 (28.6%)
Weight loss	1 (4.8%)

Upon admission, one patient presented with anemia (Hemoglobin: 68 g/L), and the others had normal hemoglobin levels. Furthermore, five patients had a high white blood cell count (> 12*10 ^ 9/L); seven, a high neutrophil count (> 75%); and four, a high C-reactive protein level (> 8 mg/L).

Sixteen patients(76.2%) underwent gastroduodenoscopy. Among them, 15 presented with gastric trichobezoars. Although the remaining one patient had gastric trichobezoar on gastroscopy performed at a local hospital 2 months back, the current examination results were normal.

**Fig. 1:**
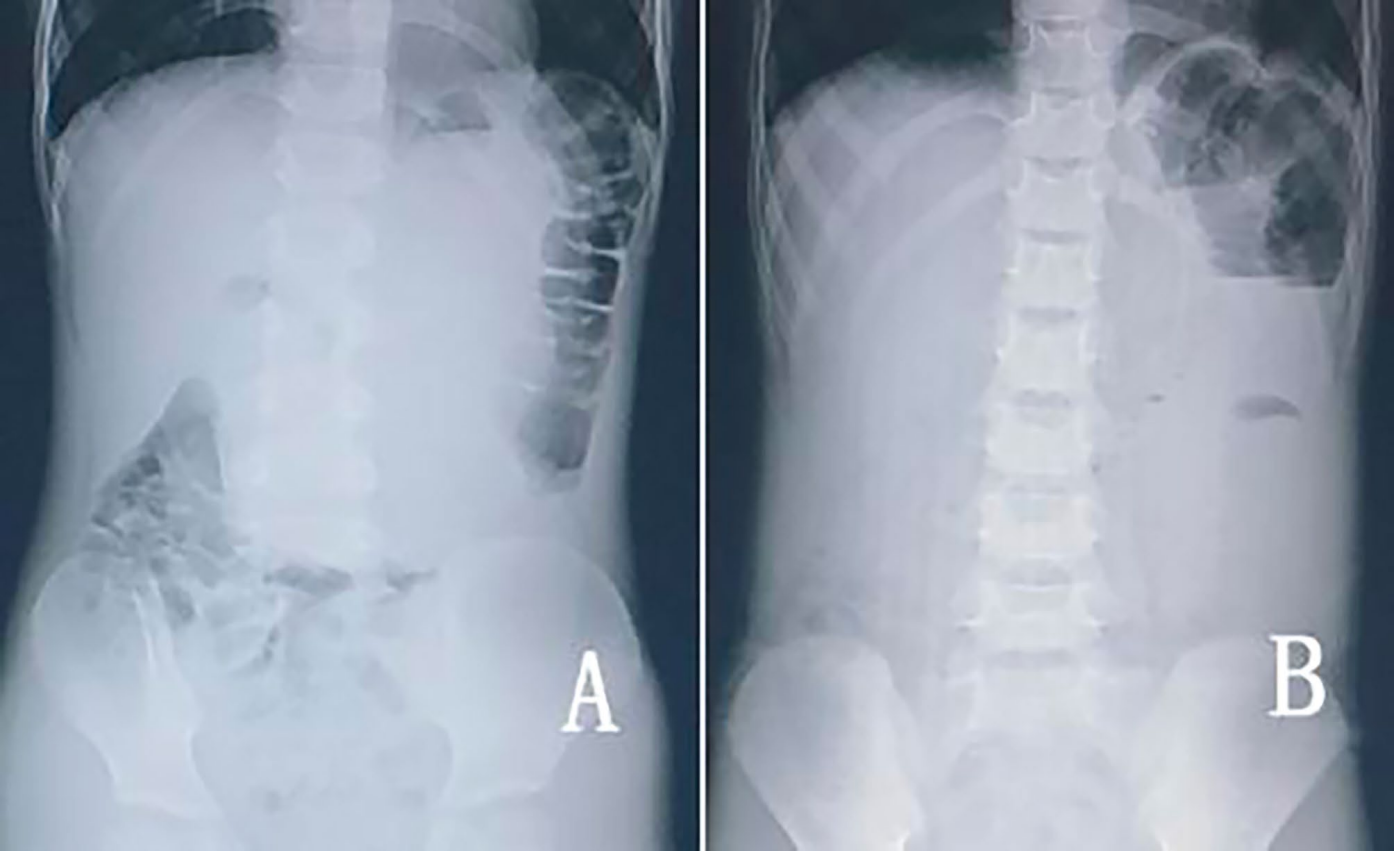
Abdominal radiography: **(A)** An epigastric mass. **(B)** Jejunal obstruction

Plain abdominal films were performed on seven patients. Results showed that two (28.6%) patients presented with left epigastric mass (Fig. 1A), three (42.9%) with small intestinal obstruction (Fig. 1B), one (14.3%) with air fluid levels, and one (14.3%) with uneven inflation.

**Fig. 2:**
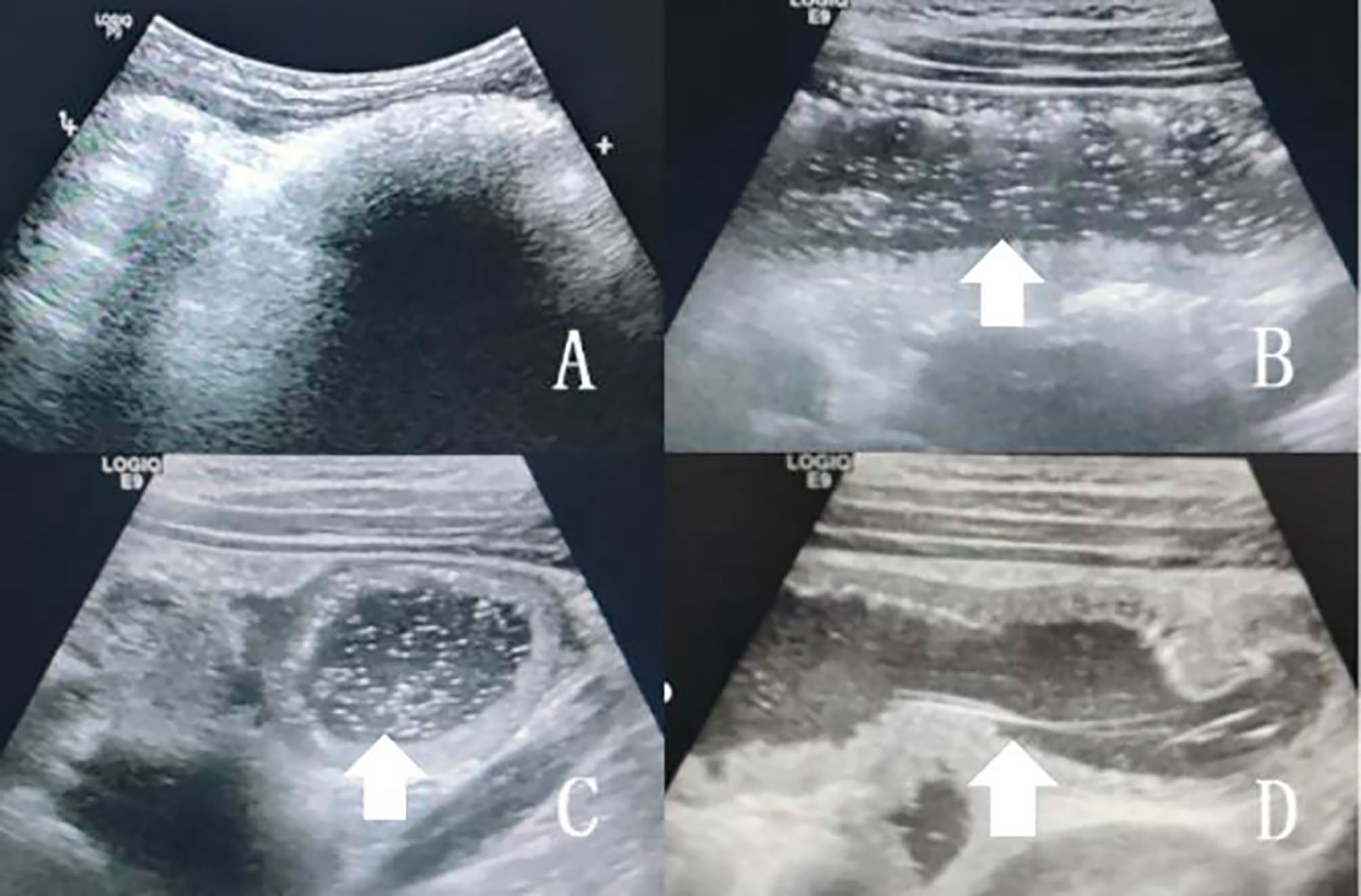
**(A)** Ultrasonography showing a hyperechoic arc-like surface of the gastric mass. **(B, C)** Multiple punctate hyperechoic points and intestinal wall thickening on the longitudinal axis and cross section of the bowel. **(D)** Fine filamentous hyperechoic structure in the longitudinal intestinal cavity

Six patients underwent ultrasonography. Results showed that two (33.3%) patients presented with hyperechogenous gastric mass (Fig. 2A), two (33.3%) with hyperechogenous small intestinal mass (Fig. 2B, C), with intestinal wall thickening, a large volume of abdominal fluid (6.5–7 cm), one (16.7%) with two concentric circles in the left midabdomen, and one (16.7%) with intussusception. A patient with small intestinal trichobezoar had a fine filamentous hyperechoic structure (Fig. 2D).

**Fig. 3:**
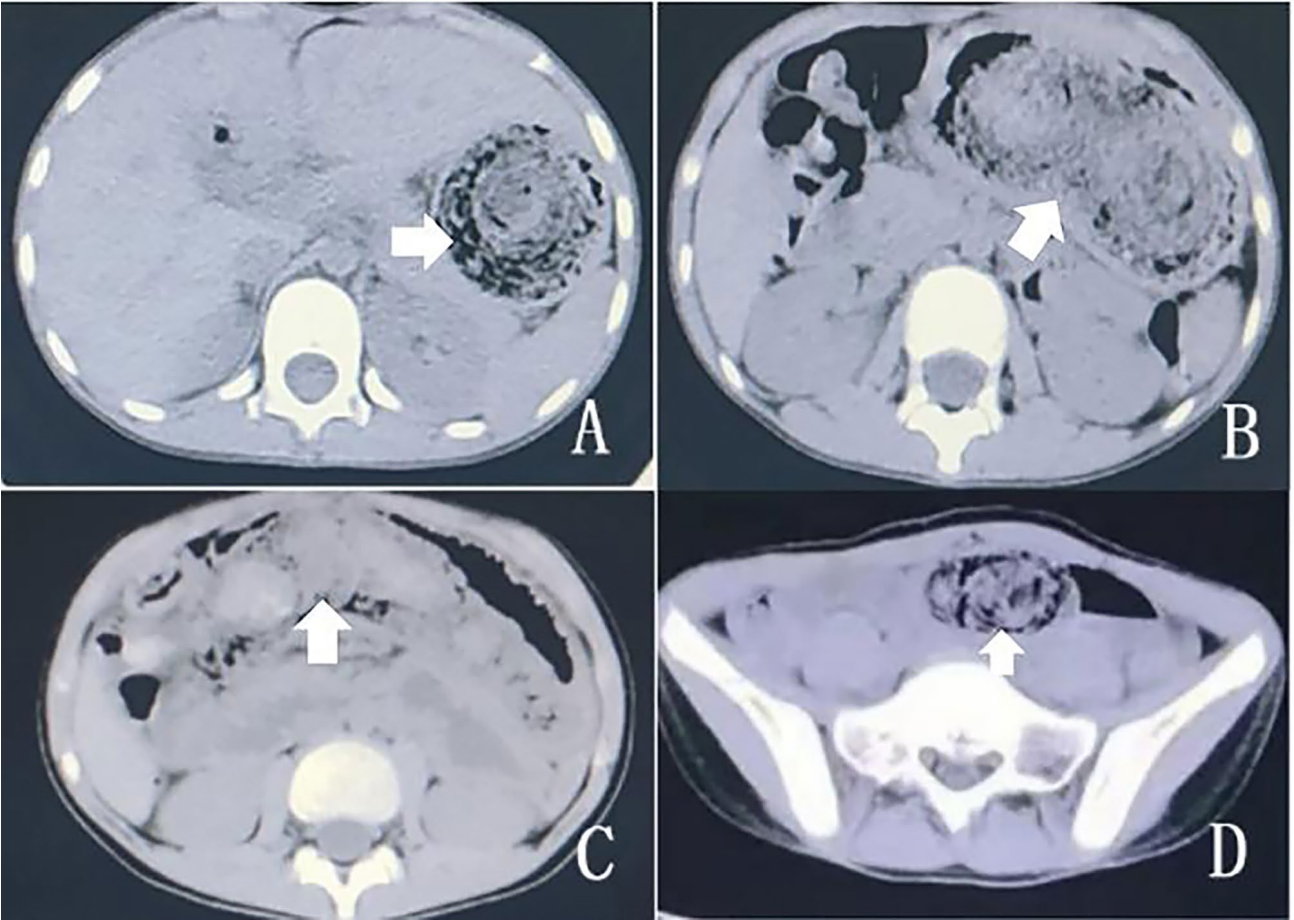
**(A, B)** Computed tomography scan of the abdomen showing intragastric BZs. **(C, D)** Small intestinal BZs.

In total, 12 patients, including 10 with gastric trichobezoars and two with small intestinal trichobezoars, underwent computed tomography (CT) scan. Nine (75%) patients presented with gastric (n = 7) and intestinal (n = 2) BZs with mixed density areas, one with abundant gastric contents, one with mild small intestinal dilatation, and one with normal findings (Fig. 3). Two patients underwent the barium contrast, and one patient presented with gastric BZ.

All patients underwent surgery, except for one whose parents refused surgery and she.

was discharged after her symptoms improved. Laparotomy was performed on 15 patients and laparoscopy-assisted minilaparotomy on five. During surgery, 10 (50%) patients were found to have gastric trichobezoar; 7 (35%), Rapunzel syndrome; and 3 (15%), isolated small bowel trichobezoars (jejunum in two patients and ileum in one) with proximally dilated loops.

Seventeen patients with Rapunzel syndrome or gastric trichobezoars underwent gastrotomy. Enterotomy was performed on three patients with small intestinal trichobezoars (Table 2). Table 3 shows the characteristics of the patients and the outcomes of laparotomy and laparoscopic assisted minilaparotomy. Enteral feeding started at an average of 6.3 ± 1.5 days. The mean postoperative hospital stay was 11 ± 2.3 (range: 7–16) days.

**Table 2:** Operative findings and surgical methods

Operative findings	Surgical methods	N (%)
Isolated gastric trichobezoar	Laparotomy, Gastrotomy	8 (40%)
	LAML, Gastrotomy	2 (10%)
Rapunzel syndrome	Laparotomy, Gastrotomy	5 (25%)
	LAML, Gastrotomy	2 (10%)
Isolated small-bowel trichobezoar	Laparotomy, Enterotomy	1 (5%)
	LAML, Enterotomy	2 (10%)

**Table 3:** Characteristics of the patients and outcomes of laparotomy and laparoscopic-assisted minilaparotomy

Variables	Laparotomy, n = 14	Laparoscopic-assisted minilaparotomy, n = 6	*P* value
Age [years, M(P25,P75)]	8.7 (7.9–10.4)	9.9 (7.7–11.1)	0.312
Body weight [kg, M(P25,P75)]	24.1 (22.0-28.9)	25.3 (21.5–42.3)	0.904
Gastrotomy: Enterotomy	13:1	2:1	0.331
Operative time [minutes, M(P25,P75)]	69.0 (57.8–92.3)	61.0 (56.8–71.8)	0.312
Intraoperative blood loss [mL, M(P25,P75)]	3.0 (2.0–5.0)	2.0 (1.75-4.0)	0.109
Incision length [cm, M(P25,P75)]	6.0 (4.75-7.0)	3.25 (2.75-5.0)	0.002*
Intraoperative complications (%)	0	0	-
Postoperative complications (%)	7.14	16.67	0.521
Fasting time [days, M(P25,P75)]	7.0 ( 5.0–8.0)	5.0 (4.75-7.0)	0.109
Hospital stay [days, M(P25,P75)]	11.5 (9.75–12.25)	10.0 (8.75–12.3)	0.444

Data are expressed as median (interquartile range) or percentage

By non-parametric test, fisher test

* *P* < 0.05

Two patients developed superficial wound infection postoperatively, which was managed with wound dressings. All patients survived, and they were then discharged. One patient developed recurrent trichobezoar 2 years after the initial trichobezoar removal. Characteristics of all patients with gastrointestinal trichobezoars are showed in Table 4.

**Table 4:** Characteristics of patients with gastrointestinal trichobezoars

Case number	Gender	Age (years)	Clinical presentation	Abdominal imaging examinations					Operation	Complications	Result
				PAF	US	CT	GDS	BC			
1	F	5.9	PM	—	—	—	Gastric trichobezoar	—	LAML, G	SWI	Cure
2	F	8.3	AP, V, PM, AD, C	Small intestinal obstruction	Hyperechogenous small intestinal mass	Small bowel bezoar with intestinal obstruction and ascites	—	Barium enema showed no organic disease	LAML, E	No	Cure
3	F	3.5	V, PM	—	—	Gastroduodenal bezoar	—	—	L, G	No	Cure
4	F	9.8	AP, V, PM, WL	Air-fluid levels	—	Gastric bezoar	Gastric trichobezoar	—	L, G	No	Cure
5	F	9.3	AP, V	—	—	—	Gastric trichobezoar	—	LAML, G	No	Cure
6	F	8.9	AP	—	—	—	Gastric trichobezoar	—	L, G	No	Cure
7	F	8.3	AP, V, C	—	—	—	Gastroduodenal trichobezoar	—	L, G	No	Cure
8	F	10.5	AP, V,PM	—	—	Abundant gastric contents	Gastric trichobezoar (local hospital)	—	L, G	No	Cure
9	F	8.5	AP, V,PM	Left epigastric mass	Hyperechogenous gastric mass	—	—	—	L, G	No	Cure
10	F	10.8	AP, V	Small intestinal obstruction	Hyperechogenous small intestinal mass	Small bowel bezoar with intestinal obstruction	Gastric trichobezoar (2 months before admission, local hospital)	—	LAML, E	No	Cure
11	F	8.8	AP, V,PM	—	—	Intragastric mass mixed density shadow, gastric bezoar	Gastric trichobezoar	—	L, G	SWI	Recurrence
12	F	8.3	AP, V	—	—	Mild small intestinal dilatation (local hospital)	Gastric trichobezoar	—	L, G	No	Cure
13	F	6.8	AP, V, AD, C	Jejunal obstruction	Two concentric circles in the left midabdomen	—	—	—	L, E	No	Cure
14	F	7.7	AP, V,PM	—	Hyperechogenous gastric mass	—	Gastric trichobezoar	—	L, G	No	Cure
15	F	8	AP, C	Left epigastric mass	—	Normal (local hospital)	Gastroduodenal trichobezoar	—	L, G	No	Cure
16	F	10.3	AP, V, AD, C	Uneven inflation	—	Intragastric mass mixed density shadow with barium residue, gastric bezoar	—	Barium swallow test showed filling defect in gastric cavity, gastric trichobezoar?	L, G	No	Cure
17	F	10.8	AP, V, PM, AD	—	—	Intragastric mass mixed density shadow, gastric bezoar	Gastric trichobezoar	—	L, G	No	Cure
18	F	10.5	AP, V, PM, C	—	—	—	Gastric trichobezoar	—	LAML, G	No	Cure
19	F	11.9	AP, PM	—	—	Intragastric mass mixed density shadow, gastric bezoar	Gastric trichobezoar	—	LAML, G	No	Cure
20	F	10.6	AP, V, PM	—	Intussusception	—	Gastric trichobezoar	—	L, G	No	Cure
21	F	8.8	AP, PM	—	—	Intragastric mass mixed density shadow, gastric bezoar	Gastric trichobezoar	—	No	—	Symptoms improved

Abbreviations: PAF plain abdominal film, US ultrosound, CT computed tomography, GS gastroscopy, BC barium contrast, F female, AP abdominal pain, V vomiting, PM palpable mass, AD abdominal distension, C constipation, WL weight loss, L laparotomy, G gastrotomy, E enterotomy, LAML laparoscopic-assisted minilaparotomy, SWI superficial wound infection

## Discussion

The resistance of human hair to digestion, limited peristaltic propulsion caused by its smooth surface, and pyloric valve effect contribute to hair accumulation between the gastric mucosal folds. Furthermore, the hair may assume the shape of the gastric lumen [4]. The propulsive movements of the stomach can extend a part of the trichobezoar to the small bowel, thereby causing the Rapunzel syndrome [5]. Occasionally, fragments of the hair conglomerate may become detached and migrate to the small bowel [6]. Therefore, there can be different outcomes, such as gastric trichobezoar, Rapunzel syndrome, enteral trichobezoar, or multiple. In some reports and reviews, gastric trichobezoar and Rapunzel syndrome accounted for 82–100% of all cases [7–10], and isolated intestinal trichobezoar was extremely rare. Intestinal trichobezoars are commonly impacted in the narrowest locations of the small bowel, such as the ileum, jejunal diverticulum, or at a postoperative stenosis [6, 11]. In our study, trichobezoars were observed in the stomach in 86% of the patients. Furthermore, three patients presented with isolated intestinal trichobezoars. The BZs were located in the jejunum, approximately 50 cm distal to the Treitz ligament, in two cases and in the ileum, 80 cm proximal to the ileocecal valve, in one case. A patient with jejunal trichobezoar underwent gastroscopy at a local hospital 2 months back. Examination revealed a large mass of hair and threads. At that time, the attempted removal failed, and the results of gastroscopy performed at our hospital were negative. Later surgery revealed that the whole hair conglomerate had migrated completely into the jejunum.

The symptoms and physical signs were based on the size and location of the trichobezoar and the presence of complications [7]. In the early stage, most patients may be asymptomatic. That is, they do not experience pathological signs or symptoms. If the size increases, the patient may experience abdominal pain, nausea, vomiting, bloating, early satiety, weight loss, hematemesis, diarrhea, and constipation. As obstruction progresses, post prandial vomiting and colicky abdominal pain may occur [12]. In our series, the most common presenting signs and symptoms were abdominal pain, nausea and vomiting, and palpable abdominal mass. In children with explicit hair eating, symptoms usually appear after 1–6 years. The symptoms of patients with gastric trichobezoars can be significantly relieved after vomiting and fasting and can be aggravated after eating. According to other reports, the major complications included stomach or intestinal perforation, intussusception, volvulus, obstructive jaundice, protein-losing enteropathy, pancreatitis, and even mortality [13]. In this study, three cases of intestinal trichobezoars were complicated with small intestinal obstruction, and the symptoms were abdominal pain and biliary vomiting. However, there were no other serious complications.

A history of hair eating or presence of hair in the vomit or feces is commonly helpful in diagnosing trichobezoar. Lack of a proper data on medical history may affect the identification of a preoperative diagnosis. In our series, the parents of some patients (n = 15) provided us with data regarding a history of hair eating or presence of hair in the vomit or feces. Therefore, the possibility of trichobezoar should be considered initially. However, the diagnosis of trichobezoar is still dependent on abdominal radiological examinations.

Gastroscopy is the gold standard for diagnosing gastric trichobezoar [14]. Under gastroscopy, direct observation is possible to obtain a clear diagnosis. However, it may not confirm the presence of a co-existing Rapunzel syndrome duo to a large mass, nor is it suitable for intestinal trichobezoar.

Plain abdominal film can show a gastric-shaped, partly opacified area. However, this condition is more helpful in diagnosing gastrointestinal obstruction or perforation [15].

BZ appears on ultrasonography as a gastric or intraluminal mass with a hyperechoic arc-like surface accompanied by acoustic shadowing [15]. Ultrasonography shows a band of increased echogenicity caused by the intermixed of hair, air, and food in the trichobezoar [14]. In our series, gastric trichobezoar present with a huge hyperechoic mass accompanied by acoustic shadowing in the back. Small intestinal trichobezoar showed multiple punctate and filamentous high-echo along the longitudinal and transverse axis of the bowel, which were more helpful for diagnosis. In some children with Rapunzel syndrome, the hair bundle passes through the pylorus on ultrasonography, and it can be differentiated from some hyperechoic tumors [9]. However, B-ultrasonography has a strong personal subjectivity. If physicians have an insufficient understanding of this disease, it can be easily misdiagnosed.

**Fig. 4:**
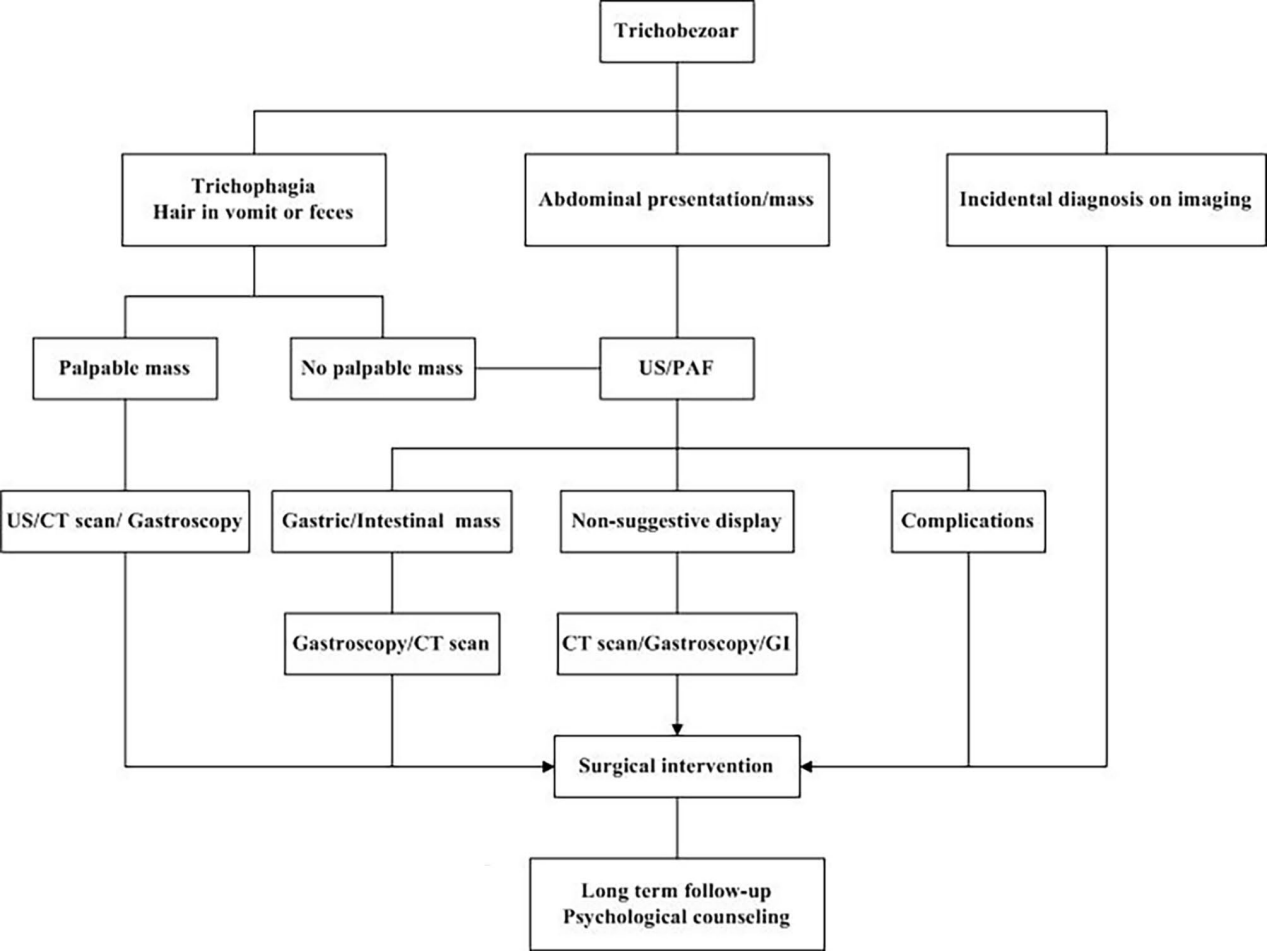
Suggested algorithm for children with Trichobezoar. Abbreviations: PAF plain abdominal film, US ultrosound, CT computed tomography, GI gastrointestinal contrast

CT scan is a superior imaging tool as it does not only identify a heterogenous BZ but can also define its extension and detect concomitant gastric and one or more small-bowel BZs [16, 17]. On CT scan image, gastric trichobezoar presents as a hypodense and heterogeneous mass within the stomach with a mesh-like pattern. Enteric BZ is a dilated small-bowel loop and a well-defined round or ovoid, heterogeneous intraluminal mass at the transition zone. Small-bowel BZs may be similar to small-bowel feces. Feces-like materials occupy a longer dilated intestinal segment, and they are located proximal to the obstruction site [15]. Our study found that gastric trichobezoars were similar, as described above, with a growth ring appearance. However, the appearance of the small intestine differed, and it was indeed similar to feces. In two patients with small-bowel trichobezoar, CT scans showed evident intraluminal masses containing patchy gas and echogenicity similar to feces, thereby occupying a longer dilated intestinal segment. The proximal intestinal wall was significantly thickened, and the distal end collapsed. Further, both structures were accompanied by ascites. However, the feces are commonly located in the colon. If observed in the small intestine, it is considered abnormal. Due to the rarity of this disease and insufficient understanding among physicians, the condition was misdiagnosed as normal gastric contents or feces in four patients in this series. Obviously, not every patient needs all these examinations. We formulated a simple algorithm which can help select an appropriate diagnostic examinations based on the history and clinical presentation (Fig. 4).

Trichobezoar should be treated to completely remove the mass and prevent recurrence. The treatment is based on the size and location of the trichobezoar, and three techniques, including laparotomy, laparoscopy, and endoscopy, have been proposed. Trichobezoar can be removed endoscopically. However, the success rate is low due to the size, density, and hardness of trichobezoar. Moreover, multiple repeated introductions of the endoscope, pressure ulceration, esophagitis, and even esophageal perforation may occur. The fragments of a large trichobezoar might migrate after fragmentation or repeated manipulation via the pylorus, thereby causing intestinal obstruction further distally [6]. In this study, in one patient, gastric trichobezoar was not successfully removed under gastroscopy at a local hospital, and intestinal obstruction developed after 2 months. The BZ was found in the jejunum. Thus, the endoscopic removal of large symptomatic trichobezoars is not appropriate, and surgery should be considered.

Due to a high success rate, low complication rate, decreased complexity, and ability to examine the whole GIT, laparotomy is still considered as the treatment of choice for trichobezoar [6]. Some reports have indicated the successful laparoscopic removal of trichobezoars in pediatric patients [18–20]. However, the procedure could prolong operative time, increase the risk of trichobezoar content spillage, and extend umbilical incision or add another incision [14]. Laparotomy was performed successfully in all cases. In five cases, LAML was conducted to remove trichobezoars. Laparoscopy can explore the stomach and small intestine to determine the location of the trichobezoar, whether there are satellite lesions or multiple trichobezoars. Thus, large incisions can be prevented, and the intestines outside the abdominal cavity will not be exposed. If the location of the trichobezoar was identified laparoscopically, a mini transverse incision was made in the left upper or right abdomen. LAML had a slight advantage over laparotomy in terms of operative time, time to initial oral feeding, and length of postoperative stay. However, the results did not significantly differ, and the incision length was significantly shorter. Minilaparotomy can remove the BZ without prolonging operative time, with a better postoperative cosmesis and shorter recovery time.

Long term follow-up and professional psychotherapy are critical after surgery to prevent recurrences [14]. However, this is not applicable to all patients who may not require psychotherapy after stopping trichophagia for several years but still need follow-up. Moreover, we had a patient with recurrent gastric trichobezoar who refused psychotherapy after surgery. Thus, trichophagia persisted, and trichobezoar formed again after 2 years.

The limitations of this study are its retrospective nature. The study was conducted at a single institution, with a small sample size, a large time span, and the heterogeneity in surgical skills among surgeons. The surgery was performed by different surgeons, and the methods were based on the surgeon’s preferences and operative experiences. Therefore, there are limitations in accurately comparing the results of patients between different surgical groups. Furthermore, larger multi-center prospective studies with standardized protocols are recommended to elucidate the role of US and CT in the diagnosis of trichobezoar, and laparoscopic surgery in the management of trichobezoar. However, to the best of our knowledge, this report is one of the largest pediatric series from a single center to date, providing evidence for the diagnosis and treatment of trichobezoar to date.

## Conclusion

Trichobezoar should be considered in young girls with a history of hair eating or those with hair in the vomit or feces. Gastroduodenoscopy is the gold standard for diagnosing gastric trichobezoar. Timely diagnosis and aggressive treatment are the keys to reducing complications and improving prognosis. LAML is a safe, feasible, and effective surgical method for treating trichobezoars.

## Data Availability

The data are not publicly available because they contain information that could compromise the privacy of research participants. Data is available from the corresponding author upon reasonable request.

## Abbreviations

GIT: gastrointestinal tract
BZ: Bezoar
CT: computed tomography
LAML: laparoscopic-assisted minilaparotomy
